# [*N*,*N*′-Bis(3-meth­oxy-2-oxidobenzyl­idene)ethane-1,2-diaminium-κ^4^ *O*,*O*′,*O*′′,*O*′′′]tris­(nitrato-κ^2^ *O*,*O*′)erbium(III)

**DOI:** 10.1107/S1600536809055184

**Published:** 2010-01-09

**Authors:** Ting Gao, Guang-Ming Li, Po Gao, Peng-Fei Yan, Guang-Feng Hou

**Affiliations:** a School of Chemistry and Materials Science, Heilongjiang University, Harbin 150080, People’s Republic of China

## Abstract

In the mononuclear salen-type complex, [Er(NO_3_)_3_(C_18_H_20_N_2_O_4_)], the Er^III^ ion is ten-coordinated in a distorted hexa­deca­hedral geometry by six O atoms of three nitrate anions and four O atoms of the salen-like ligand. Inter­molecular N—H⋯O hydrogen bonds occur. The crystal structure is stabilized by inter­molecular C—H⋯O hydrogen bonds.

## Related literature

For similar lanthanide complexes of the same salen-like ligand, see: Gao *et al.* (2008 ▶, 2009 ▶).
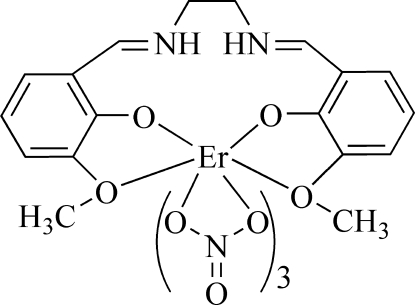


## Experimental

### 

#### Crystal data

[Er(NO_3_)_3_(C_18_H_20_N_2_O_4_)]
*M* *_r_* = 681.65
Monoclinic, 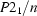

*a* = 14.098 (3) Å
*b* = 11.865 (2) Å
*c* = 14.571 (3) Å
β = 103.98 (3)°
*V* = 2365.1 (8) Å^3^
*Z* = 4
Mo *K*α radiation
μ = 3.63 mm^−1^
*T* = 291 K
0.37 × 0.36 × 0.34 mm

#### Data collection

Rigaku R-AXIS RAPID diffractometer
Absorption correction: multi-scan (*ABSCOR*; Higashi, 1995 ▶) *T*_min_ = 0.344, *T*_max_ = 0.368
21792 measured reflections
5358 independent reflections
4748 reflections with *I* > 2σ(*I*)
*R*_int_ = 0.024

#### Refinement

*R*[*F* ^2^ > 2σ(*F* ^2^)] = 0.022
*wR*(*F* ^2^) = 0.049
*S* = 1.04
5358 reflections
344 parameters
2 restraints
H atoms treated by a mixture of independent and constrained refinement
Δρ_max_ = 0.97 e Å^−3^
Δρ_min_ = −0.32 e Å^−3^

### 

Data collection: *RAPID-AUTO* (Rigaku, 1998 ▶); cell refinement: *RAPID-AUTO*; data reduction: *CrystalClear* (Rigaku/MSC, 2002 ▶); program(s) used to solve structure: *SHELXS97* (Sheldrick, 2008 ▶); program(s) used to refine structure: *SHELXL97* (Sheldrick, 2008 ▶); molecular graphics: *SHELXTL* (Sheldrick, 2008 ▶); software used to prepare material for publication: *SHELXL97*.

## Supplementary Material

Crystal structure: contains datablocks global, I. DOI: 10.1107/S1600536809055184/pk2218sup1.cif

Structure factors: contains datablocks I. DOI: 10.1107/S1600536809055184/pk2218Isup2.hkl

Additional supplementary materials:  crystallographic information; 3D view; checkCIF report

## Figures and Tables

**Table 1:** Hydrogen-bond geometry (Å, °)

*D*—H⋯*A*	*D*—H	H⋯*A*	*D*⋯*A*	*D*—H⋯*A*
N2—H2⋯O3	0.85 (1)	1.89 (3)	2.574 (3)	137 (3)
N1—H1⋯O1	0.85 (1)	1.84 (2)	2.567 (3)	143 (3)
C7—H7⋯O5^i^	0.93	2.33	3.073 (3)	137
C9—H9*A*⋯O12^ii^	0.97	2.50	3.241 (3)	133
C10—H10⋯O9^iii^	0.93	2.57	3.395 (4)	148
C14—H14⋯O12^iv^	0.93	2.50	3.341 (4)	150

Symmetry codes: (i) 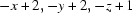; (ii) 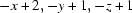; (iii) 
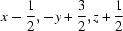
; (iv) 
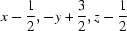
.

**Fig. 1.**
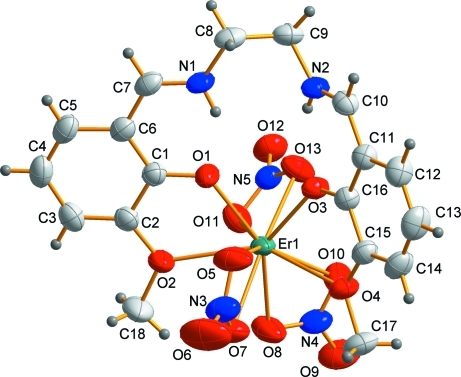
The molecular structure of the the title compound, showing 30% probability displacement ellipsoids.

## supplementary crystallographic information

### Comment

In continuation of our studies of salen-type lanthanide complexes (Gao *et al.*, 2008, 2009), we present here the crystal structure of the title compound. As shown in Fig. 1, the ten-coordinate Er^III^ ion adopts a hexadecahedral geometry provided by the O atoms of three bidentate nitrate anions and by one ligand that utilizes two hydroxyl and two methoxy oxygen atoms, while the protonated nitrogen atoms remain uncoordinated. This compound is isostructural with the corresponding Nd, Eu, Tb and Dy complexes (Gao *et al.*, 2008, 2009). The Er—O bond distances range from 2.2462 (19) to 2.682 (2) Å, with the shorter bonds involving the O1 and O3 deprotonated phenol oxygen atoms. The crystal structure is stabilized by intra- and intermolecular N—H···O and C—H···O hydrogen bonds (Table 1).

### Experimental

The title complex was obtained by the treatment of erbium (III) nitrate hexahydrate (0.114 g, 0.25 mmol) with the salen-type ligand (0.083 g, 0.25 mmol) in acetonitrile/methanol (10 ml/10 ml). The mixture was stirred for 3 h. The reaction mixture was filtered; diethyl ether was allowed to diffuse slowly into the solution of the filtrate. Single crystals were obtained after several days. Analysis calculated for for C_18_H_20_Er_1_N_5_O_13_: C 31.72, H 2.96, N 10.27%; found: C 32.08, H 3.00, N 10.38%.

### Refinement

H atoms bound to C atoms were placed in calculated positions and treated as riding on their parent atoms, with C—H = 0.93 Å (aromatic C), C—H = 0.97Å (methylene C), and with *U*_iso_(H) = 1.2Ueq(C) or C—H = 0.96 Å (methyl C) and with *U*_iso_(H) = 1.5Ueq(C). The N-bound H atoms were initially located in a difference Fourier map and they were refined with the N—H bond distance restrained to 0.85 Å.

### Crystal data

**Table:**

[Er(NO_3_)_3_(C_18_H_20_N_2_O_4_)]	*F*(000) = 1340
*M**_r_* = 681.65	*D*_x_ = 1.914 Mg m^−^^3^
Monoclinic, *P*2_1_/*n*	Mo *K*α radiation, λ = 0.71073 Å
Hall symbol: -P 2yn	Cell parameters from 19557 reflections
*a* = 14.098 (3) Å	θ = 6.7–55.0°
*b* = 11.865 (2) Å	µ = 3.63 mm^−^^1^
*c* = 14.571 (3) Å	*T* = 291 K
β = 103.98 (3)°	Block, brown
*V* = 2365.1 (8) Å^3^	0.37 × 0.36 × 0.34 mm
*Z* = 4	

### Data collection

**Table:**

Rigaku R-AXIS RAPID diffractometer	5358 independent reflections
Radiation source: fine-focus sealed tube	4748 reflections with *I* > 2σ(*I*)
graphite	*R*_int_ = 0.024
Detector resolution: 10.000 pixels mm^-1^	θ_max_ = 27.5°, θ_min_ = 3.4°
ω scans	*h* = −18→18
Absorption correction: multi-scan (*ABSCOR*; Higashi, 1995)	*k* = −15→13
*T*_min_ = 0.344, *T*_max_ = 0.368	*l* = −18→18
21792 measured reflections	

### Refinement

**Table:**

Refinement on *F*^2^	Primary atom site location: structure-invariant direct methods
Least-squares matrix: full	Secondary atom site location: difference Fourier map
*R*[*F*^2^ > 2σ(*F*^2^)] = 0.022	Hydrogen site location: inferred from neighbouring sites
*wR*(*F*^2^) = 0.049	H atoms treated by a mixture of independent and constrained refinement
*S* = 1.04	*w* = 1/[σ^2^(*F*_o_^2^) + (0.0212*P*)^2^ + 1.495*P*] where *P* = (*F*_o_^2^ + 2*F*_c_^2^)/3
5358 reflections	(Δ/σ)_max_ = 0.002
344 parameters	Δρ_max_ = 0.97 e Å^−^^3^
2 restraints	Δρ_min_ = −0.32 e Å^−^^3^

### Special details

**Table:**

Geometry. All e.s.d.'s (except the e.s.d. in the dihedral angle between two l.s. planes) are estimated using the full covariance matrix. The cell e.s.d.'s are taken into account individually in the estimation of e.s.d.'s in distances, angles and torsion angles; correlations between e.s.d.'s in cell parameters are only used when they are defined by crystal symmetry. An approximate (isotropic) treatment of cell e.s.d.'s is used for estimating e.s.d.'s involving l.s. planes.
Refinement. Refinement of *F*^2^ against ALL reflections. The weighted *R*-factor *wR* and goodness of fit *S* are based on *F*^2^, conventional *R*-factors *R* are based on *F*, with *F* set to zero for negative *F*^2^. The threshold expression of *F*^2^ > σ(*F*^2^) is used only for calculating *R*-factors(gt) *etc*. and is not relevant to the choice of reflections for refinement. *R*-factors based on *F*^2^ are statistically about twice as large as those based on *F*, and *R*- factors based on ALL data will be even larger.

### Fractional atomic coordinates and isotropic or equivalent isotropic displacement parameters (Å^2^)

**Table:**

	*x*	*y*	*z*	*U*_iso_*/*U*_eq_	
C1	1.11705 (18)	0.8605 (2)	0.45224 (18)	0.0382 (5)	
C2	1.20341 (19)	0.8854 (2)	0.42275 (19)	0.0407 (5)	
C3	1.28633 (19)	0.9208 (2)	0.4865 (2)	0.0473 (6)	
H3	1.3434	0.9334	0.4667	0.057*	
C4	1.2851 (2)	0.9379 (2)	0.5812 (2)	0.0514 (7)	
H4	1.3416	0.9618	0.6241	0.062*	
C5	1.2025 (2)	0.9199 (2)	0.6108 (2)	0.0482 (6)	
H5	1.2021	0.9338	0.6736	0.058*	
C6	1.11664 (18)	0.8802 (2)	0.54721 (17)	0.0398 (5)	
C7	1.03136 (19)	0.8571 (2)	0.57937 (18)	0.0419 (6)	
H7	1.0340	0.8661	0.6433	0.050*	
C8	0.8585 (2)	0.8013 (2)	0.5501 (2)	0.0476 (6)	
H8A	0.8190	0.8692	0.5424	0.057*	
H8B	0.8721	0.7791	0.6161	0.057*	
C9	0.8026 (2)	0.7084 (2)	0.4892 (2)	0.0463 (6)	
H9A	0.8384	0.6381	0.5029	0.056*	
H9B	0.7395	0.6985	0.5038	0.056*	
C10	0.71313 (18)	0.7852 (2)	0.33653 (19)	0.0405 (6)	
H10	0.6585	0.7963	0.3607	0.049*	
C11	0.70956 (17)	0.8234 (2)	0.24387 (18)	0.0378 (5)	
C12	0.62437 (19)	0.8736 (2)	0.1881 (2)	0.0487 (6)	
H12	0.5690	0.8799	0.2118	0.058*	
C13	0.6223 (2)	0.9123 (3)	0.1009 (2)	0.0562 (8)	
H13	0.5652	0.9442	0.0647	0.067*	
C14	0.7057 (2)	0.9052 (2)	0.0634 (2)	0.0509 (7)	
H14	0.7033	0.9319	0.0029	0.061*	
C15	0.78977 (18)	0.8588 (2)	0.11660 (18)	0.0393 (5)	
C16	0.79432 (17)	0.8157 (2)	0.20754 (18)	0.0353 (5)	
C17	0.8799 (3)	0.8857 (3)	−0.0015 (2)	0.0660 (9)	
H15	0.8287	0.8495	−0.0475	0.099*	
H16	0.9420	0.8669	−0.0135	0.099*	
H17	0.8708	0.9659	−0.0056	0.099*	
C18	1.2751 (3)	0.8925 (4)	0.2892 (3)	0.0813 (12)	
H18	1.2967	0.9685	0.3043	0.122*	
H19	1.2567	0.8835	0.2217	0.122*	
H20	1.3270	0.8411	0.3158	0.122*	
Er1	1.027172 (8)	0.791612 (9)	0.235283 (7)	0.03487 (4)	
H1	0.954 (2)	0.824 (3)	0.4667 (10)	0.055 (9)*	
H2	0.8358 (18)	0.723 (3)	0.364 (2)	0.065 (10)*	
N1	0.95017 (17)	0.8241 (2)	0.52382 (16)	0.0431 (5)	
N2	0.78847 (16)	0.7357 (2)	0.38932 (16)	0.0410 (5)	
N3	1.0155 (2)	1.0328 (2)	0.1988 (2)	0.0662 (7)	
N4	1.07427 (19)	0.6872 (2)	0.07369 (18)	0.0498 (6)	
N5	1.08572 (16)	0.57223 (19)	0.31635 (15)	0.0422 (5)	
O1	1.04131 (13)	0.82028 (18)	0.39117 (13)	0.0500 (5)	
O2	1.19230 (13)	0.86931 (18)	0.32755 (13)	0.0500 (5)	
O3	0.87510 (12)	0.77016 (15)	0.25531 (12)	0.0393 (4)	
O4	0.87710 (14)	0.84819 (17)	0.09111 (13)	0.0485 (4)	
O5	0.97238 (19)	0.9849 (2)	0.25442 (16)	0.0729 (7)	
O6	1.0101 (3)	1.1337 (2)	0.1861 (3)	0.1093 (11)	
O7	1.06391 (17)	0.96856 (19)	0.15897 (16)	0.0613 (6)	
O8	1.12293 (19)	0.7666 (2)	0.11639 (19)	0.0714 (7)	
O9	1.0911 (2)	0.6461 (2)	0.00302 (18)	0.0794 (7)	
O10	1.00528 (15)	0.6536 (2)	0.10706 (16)	0.0615 (6)	
O11	1.14616 (13)	0.64176 (18)	0.29752 (16)	0.0543 (5)	
O12	1.11095 (15)	0.48027 (17)	0.35168 (15)	0.0566 (5)	
O13	0.99850 (14)	0.60343 (17)	0.29684 (16)	0.0547 (5)	

### Atomic displacement parameters (Å^2^)

**Table:**

	*U*^11^	*U*^22^	*U*^33^	*U*^12^	*U*^13^	*U*^23^
C1	0.0425 (13)	0.0366 (14)	0.0352 (13)	−0.0009 (10)	0.0089 (10)	0.0014 (10)
C2	0.0466 (14)	0.0346 (13)	0.0415 (14)	−0.0049 (10)	0.0119 (10)	−0.0005 (11)
C3	0.0432 (14)	0.0371 (14)	0.0607 (18)	−0.0041 (11)	0.0111 (12)	−0.0004 (13)
C4	0.0519 (16)	0.0423 (16)	0.0522 (17)	−0.0049 (12)	−0.0025 (12)	−0.0064 (13)
C5	0.0614 (17)	0.0391 (15)	0.0390 (15)	0.0005 (12)	0.0023 (12)	−0.0049 (11)
C6	0.0511 (14)	0.0329 (13)	0.0347 (13)	0.0027 (10)	0.0092 (10)	−0.0002 (10)
C7	0.0570 (16)	0.0376 (14)	0.0321 (13)	0.0075 (11)	0.0127 (11)	−0.0027 (10)
C8	0.0512 (15)	0.0602 (18)	0.0377 (14)	0.0090 (12)	0.0229 (11)	0.0051 (12)
C9	0.0495 (15)	0.0522 (16)	0.0425 (15)	0.0046 (11)	0.0217 (11)	0.0143 (12)
C10	0.0382 (13)	0.0407 (14)	0.0461 (15)	−0.0002 (10)	0.0170 (10)	−0.0042 (11)
C11	0.0364 (12)	0.0369 (13)	0.0406 (14)	0.0045 (9)	0.0102 (10)	−0.0024 (10)
C12	0.0402 (14)	0.0496 (16)	0.0546 (17)	0.0106 (11)	0.0084 (11)	−0.0028 (13)
C13	0.0496 (16)	0.0533 (18)	0.0577 (19)	0.0179 (13)	−0.0027 (13)	0.0056 (14)
C14	0.0638 (18)	0.0448 (16)	0.0390 (15)	0.0085 (12)	0.0022 (12)	0.0072 (12)
C15	0.0460 (14)	0.0371 (14)	0.0347 (13)	0.0039 (10)	0.0094 (10)	0.0018 (10)
C16	0.0378 (12)	0.0328 (13)	0.0353 (13)	0.0031 (9)	0.0091 (9)	0.0009 (9)
C17	0.081 (2)	0.084 (2)	0.0378 (16)	0.0008 (18)	0.0249 (15)	0.0118 (16)
C18	0.068 (2)	0.121 (3)	0.066 (2)	−0.041 (2)	0.0381 (18)	−0.012 (2)
Er1	0.03856 (7)	0.04067 (7)	0.02990 (6)	−0.00224 (4)	0.01707 (4)	0.00026 (4)
N1	0.0496 (13)	0.0531 (14)	0.0309 (12)	0.0030 (10)	0.0181 (9)	0.0011 (10)
N2	0.0410 (12)	0.0482 (13)	0.0382 (12)	0.0027 (9)	0.0183 (9)	0.0038 (9)
N3	0.107 (2)	0.0498 (17)	0.0492 (15)	−0.0087 (14)	0.0328 (15)	−0.0004 (13)
N4	0.0628 (15)	0.0502 (15)	0.0439 (13)	0.0119 (11)	0.0270 (11)	0.0006 (11)
N5	0.0452 (12)	0.0476 (13)	0.0352 (11)	0.0032 (9)	0.0124 (9)	0.0028 (9)
O1	0.0430 (10)	0.0761 (14)	0.0332 (10)	−0.0144 (9)	0.0136 (8)	−0.0086 (9)
O2	0.0476 (10)	0.0648 (13)	0.0430 (11)	−0.0166 (9)	0.0215 (8)	−0.0047 (9)
O3	0.0348 (9)	0.0484 (11)	0.0360 (9)	0.0081 (7)	0.0109 (7)	0.0099 (7)
O4	0.0574 (11)	0.0581 (12)	0.0335 (10)	0.0032 (9)	0.0179 (8)	0.0075 (8)
O5	0.127 (2)	0.0497 (14)	0.0611 (15)	−0.0098 (12)	0.0588 (15)	−0.0098 (10)
O6	0.192 (3)	0.0431 (16)	0.116 (3)	0.0054 (17)	0.082 (2)	0.0152 (16)
O7	0.0805 (15)	0.0568 (13)	0.0560 (13)	−0.0025 (11)	0.0344 (11)	0.0099 (11)
O8	0.0880 (17)	0.0701 (16)	0.0739 (17)	−0.0253 (13)	0.0539 (14)	−0.0199 (13)
O9	0.118 (2)	0.0705 (17)	0.0685 (16)	0.0085 (14)	0.0590 (15)	−0.0145 (13)
O10	0.0520 (12)	0.0793 (16)	0.0589 (14)	−0.0083 (10)	0.0245 (10)	−0.0197 (11)
O11	0.0397 (10)	0.0558 (13)	0.0686 (14)	−0.0013 (8)	0.0151 (9)	0.0030 (10)
O12	0.0679 (13)	0.0489 (12)	0.0524 (12)	0.0159 (9)	0.0132 (9)	0.0122 (10)
O13	0.0414 (10)	0.0518 (12)	0.0753 (14)	0.0058 (8)	0.0225 (9)	0.0181 (10)

### Geometric parameters (Å, °)

**Table:**

C1—O1	1.304 (3)	C15—C16	1.407 (3)
C1—C6	1.405 (3)	C16—O3	1.300 (3)
C1—C2	1.417 (3)	C17—O4	1.430 (3)
C2—C3	1.371 (4)	C17—H15	0.9600
C2—O2	1.371 (3)	C17—H16	0.9600
C3—C4	1.399 (4)	C17—H17	0.9600
C3—H3	0.9300	C18—O2	1.437 (3)
C4—C5	1.354 (4)	C18—H18	0.9600
C4—H4	0.9300	C18—H19	0.9600
C5—C6	1.416 (4)	C18—H20	0.9600
C5—H5	0.9300	Er1—O3	2.2469 (17)
C6—C7	1.419 (4)	Er1—O1	2.2576 (19)
C7—N1	1.293 (3)	Er1—O10	2.447 (2)
C7—H7	0.9300	Er1—O5	2.458 (2)
C8—N1	1.460 (3)	Er1—O8	2.458 (2)
C8—C9	1.512 (4)	Er1—O11	2.462 (2)
C8—H8A	0.9700	Er1—O13	2.476 (2)
C8—H8B	0.9700	Er1—O7	2.488 (2)
C9—N2	1.457 (3)	Er1—O2	2.562 (2)
C9—H9A	0.9700	Er1—O4	2.682 (2)
C9—H9B	0.9700	Er1—N4	2.877 (2)
C10—N2	1.292 (3)	Er1—N5	2.895 (2)
C10—C11	1.413 (4)	N1—H1	0.846 (10)
C10—H10	0.9300	N2—H2	0.847 (10)
C11—C12	1.409 (4)	N3—O6	1.211 (4)
C11—C16	1.422 (3)	N3—O7	1.255 (3)
C12—C13	1.344 (4)	N3—O5	1.260 (3)
C12—H12	0.9300	N4—O9	1.213 (3)
C13—C14	1.413 (4)	N4—O8	1.241 (3)
C13—H13	0.9300	N4—O10	1.253 (3)
C14—C15	1.365 (4)	N5—O12	1.222 (3)
C14—H14	0.9300	N5—O13	1.249 (3)
C15—O4	1.375 (3)	N5—O11	1.263 (3)
			
O1—C1—C6	122.4 (2)	O8—Er1—O13	108.19 (8)
O1—C1—C2	119.4 (2)	O11—Er1—O13	51.07 (6)
C6—C1—C2	118.2 (2)	O3—Er1—O7	117.50 (7)
C3—C2—O2	126.7 (2)	O1—Er1—O7	110.20 (8)
C3—C2—C1	120.9 (2)	O10—Er1—O7	103.00 (8)
O2—C2—C1	112.4 (2)	O5—Er1—O7	50.82 (7)
C2—C3—C4	120.1 (3)	O8—Er1—O7	65.02 (8)
C2—C3—H3	120.0	O11—Er1—O7	125.25 (7)
C4—C3—H3	120.0	O13—Er1—O7	173.13 (7)
C5—C4—C3	120.6 (3)	O3—Er1—O2	138.34 (6)
C5—C4—H4	119.7	O1—Er1—O2	64.51 (6)
C3—C4—H4	119.7	O10—Er1—O2	124.92 (7)
C4—C5—C6	120.6 (3)	O5—Er1—O2	82.83 (8)
C4—C5—H5	119.7	O8—Er1—O2	80.16 (8)
C6—C5—H5	119.7	O11—Er1—O2	67.34 (7)
C1—C6—C5	119.6 (2)	O13—Er1—O2	109.97 (7)
C1—C6—C7	119.9 (2)	O7—Er1—O2	70.74 (7)
C5—C6—C7	120.4 (2)	O3—Er1—O4	62.17 (6)
N1—C7—C6	123.0 (2)	O1—Er1—O4	127.71 (7)
N1—C7—H7	118.5	O10—Er1—O4	68.86 (7)
C6—C7—H7	118.5	O5—Er1—O4	69.22 (8)
N1—C8—C9	110.5 (2)	O8—Er1—O4	86.66 (8)
N1—C8—H8A	109.6	O11—Er1—O4	143.86 (7)
C9—C8—H8A	109.6	O13—Er1—O4	110.04 (7)
N1—C8—H8B	109.6	O7—Er1—O4	69.43 (7)
C9—C8—H8B	109.6	O2—Er1—O4	139.98 (7)
H8A—C8—H8B	108.1	O3—Er1—N4	118.27 (8)
N2—C9—C8	110.5 (2)	O1—Er1—N4	155.06 (7)
N2—C9—H9A	109.5	O10—Er1—N4	25.63 (7)
C8—C9—H9A	109.5	O5—Er1—N4	129.48 (7)
N2—C9—H9B	109.5	O8—Er1—N4	25.37 (7)
C8—C9—H9B	109.5	O11—Er1—N4	73.69 (7)
H9A—C9—H9B	108.1	O13—Er1—N4	89.91 (7)
N2—C10—C11	123.3 (2)	O7—Er1—N4	83.31 (7)
N2—C10—H10	118.4	O2—Er1—N4	102.96 (7)
C11—C10—H10	118.4	O4—Er1—N4	76.17 (7)
C12—C11—C10	121.0 (2)	O3—Er1—N5	91.84 (6)
C12—C11—C16	119.2 (2)	O1—Er1—N5	77.16 (7)
C10—C11—C16	119.7 (2)	O10—Er1—N5	71.38 (8)
C13—C12—C11	120.7 (3)	O5—Er1—N5	149.24 (7)
C13—C12—H12	119.7	O8—Er1—N5	91.62 (8)
C11—C12—H12	119.7	O11—Er1—N5	25.67 (6)
C12—C13—C14	121.0 (2)	O13—Er1—N5	25.39 (6)
C12—C13—H13	119.5	O7—Er1—N5	150.63 (7)
C14—C13—H13	119.5	O2—Er1—N5	88.63 (7)
C15—C14—C13	119.6 (3)	O4—Er1—N5	129.63 (6)
C15—C14—H14	120.2	N4—Er1—N5	81.22 (7)
C13—C14—H14	120.2	C7—N1—C8	126.7 (2)
C14—C15—O4	126.5 (2)	C7—N1—H1	111 (2)
C14—C15—C16	121.1 (2)	C8—N1—H1	122 (2)
O4—C15—C16	112.4 (2)	C10—N2—C9	126.2 (2)
O3—C16—C15	119.3 (2)	C10—N2—H2	116 (2)
O3—C16—C11	122.2 (2)	C9—N2—H2	117 (2)
C15—C16—C11	118.4 (2)	O6—N3—O7	123.4 (3)
O4—C17—H15	109.5	O6—N3—O5	121.5 (3)
O4—C17—H16	109.5	O7—N3—O5	115.1 (3)
H15—C17—H16	109.5	O6—N3—Er1	178.1 (2)
O4—C17—H17	109.5	O7—N3—Er1	58.24 (15)
H15—C17—H17	109.5	O5—N3—Er1	56.87 (16)
H16—C17—H17	109.5	O9—N4—O8	122.3 (3)
O2—C18—H18	109.5	O9—N4—O10	122.0 (3)
O2—C18—H19	109.5	O8—N4—O10	115.7 (2)
H18—C18—H19	109.5	O9—N4—Er1	177.1 (2)
O2—C18—H20	109.5	O8—N4—Er1	58.09 (13)
H18—C18—H20	109.5	O10—N4—Er1	57.67 (14)
H19—C18—H20	109.5	O12—N5—O13	122.0 (2)
O3—Er1—O1	75.02 (7)	O12—N5—O11	122.1 (2)
O3—Er1—O10	94.30 (7)	O13—N5—O11	115.8 (2)
O1—Er1—O10	146.42 (8)	O12—N5—Er1	179.17 (19)
O3—Er1—O5	75.82 (7)	O13—N5—Er1	58.18 (13)
O1—Er1—O5	72.48 (8)	O11—N5—Er1	57.65 (13)
O10—Er1—O5	136.56 (9)	C1—O1—Er1	126.89 (16)
O3—Er1—O8	141.41 (8)	C2—O2—C18	117.5 (2)
O1—Er1—O8	142.88 (8)	C2—O2—Er1	116.64 (14)
O10—Er1—O8	51.00 (7)	C18—O2—Er1	125.79 (19)
O5—Er1—O8	115.70 (8)	C16—O3—Er1	128.54 (15)
O3—Er1—O11	117.19 (6)	C15—O4—C17	117.2 (2)
O1—Er1—O11	81.46 (8)	C15—O4—Er1	113.57 (14)
O10—Er1—O11	75.35 (8)	C17—O4—Er1	128.36 (18)
O5—Er1—O11	146.82 (8)	N3—O5—Er1	97.71 (18)
O8—Er1—O11	74.41 (9)	N3—O7—Er1	96.36 (16)
O3—Er1—O13	66.82 (6)	N4—O8—Er1	96.54 (16)
O1—Er1—O13	75.74 (8)	N4—O10—Er1	96.69 (17)
O10—Er1—O13	70.83 (8)	N5—O11—Er1	96.68 (14)
O5—Er1—O13	135.84 (7)	N5—O13—Er1	96.42 (15)

### Hydrogen-bond geometry (Å, °)

**Table:**

*D*—H···*A*	*D*—H	H···*A*	*D*···*A*	*D*—H···*A*
N2—H2···O3	0.85 (1)	1.89 (3)	2.574 (3)	137 (3)
N1—H1···O1	0.85 (1)	1.84 (2)	2.567 (3)	143 (3)
C7—H7···O5^i^	0.93	2.33	3.073 (3)	137
C9—H9A···O12^ii^	0.97	2.50	3.241 (3)	133
C10—H10···O9^iii^	0.93	2.57	3.395 (4)	148
C14—H14···O12^iv^	0.93	2.50	3.341 (4)	150

Symmetry codes: (i) −*x*+2, −*y*+2, −*z*+1; (ii) −*x*+2, −*y*+1, −*z*+1; (iii) *x*−1/2, −*y*+3/2, *z*+1/2; (iv) *x*−1/2, −*y*+3/2, *z*−1/2.

## References

[bb1] Gao, T., Li, G.-M., Gao, P., Yan, P.-F. & Hou, G.-F. (2009). *Acta Cryst.* E**65**, m1585. 10.1107/S1600536809047436 PMC2972125 21578615

[bb2] Gao, T., Yan, P. F., Li, G. M., Hou, G. F. & Gao, J. S. (2008). *Inorg. Chim. Acta*, **361**, 2051–2058.

[bb3] Higashi, T. (1995). *ABSCOR* Rigaku Corporation, Tokyo, Japan.

[bb5] Rigaku (1998). *RAPID-AUTO* Rigaku Corporation, Tokyo, Japan.

[bb6] Rigaku/MSC (2002). *CrystalClear* Rigaku/MSC Inc., The Woodlands, Texas, USA.

[bb7] Sheldrick, G. M. (2008). *Acta Cryst.* A**64**, 112–122. 10.1107/S0108767307043930 18156677

